# Prevalence of Dental Caries in Schoolchildren from the Galapagos Islands: ESSO-Gal Cohort Report

**DOI:** 10.1155/2023/6544949

**Published:** 2023-12-11

**Authors:** Ana Armas-Vega, Juan Marcos Parise-Vasco, María Cristina Díaz-Segovia, David Alejandro Arroyo-Bonilla, María José Cabrera-Dávila, María Christel Zambrano-Bonilla, Ingrid Ordonez-Romero, Andrea Caiza-Rennella, Andrea Zambrano-Mendoza, Celia Ponce-Faula, Andrés Viteri-García

**Affiliations:** ^1^Carrera de Odontología, Facultad de Ciencias de la Salud Eugenio Espejo, Universidad UTE, Quito, Ecuador; ^2^Centro de Investigación en Salud Pública y Epidemiología Clínica (CISPEC), Facultad de Ciencias de la Salud Eugenio Espejo, Universidad UTE, Quito, Ecuador; ^3^Posgrado de Odontopediatría, Facultad de Ciencias de la Salud Eugenio Espejo, Universidad UTE, Quito, Ecuador; ^4^Federal University of Rio Grande do Sul, Porto Alegre, Brazil; ^5^Facultad de Odontología, Universidad Católica de Santiago de Guayaquil, Guayaquil, Ecuador; ^6^Facultad de Odontología, Universidad de Guayaquil, Guayaquil, Ecuador; ^7^Carrera de Odontología, Universidad San Gregorio de Portoviejo, Portoviejo, Ecuador

## Abstract

**Objective:**

Dental caries remains a prevalent disease worldwide. Several epidemiological studies have shown that it affects the oral health of the pediatric population, and the Galapagos population in Ecuador is no exception. The aim of this study was to determine the prevalence of dental caries and its association, based on baseline information from the Galapagos Oral Health Study (ESSO-Gal), in children of the Galapagos Islands, Ecuador.

**Methods:**

A cross-sectional study was conducted involving 804 children aged 2–11 years. The prevalence of dental caries was assessed using the International Caries Detection and Assessment System (ICDAS II) criteria, while the presence of dental biofilm was assessed using the Silness–Löe index. Descriptive statistics, including frequency analysis and measures of central tendency and dispersion, were performed. Inferential statistical analyses were conducted to identify associations between variables. Statistical analyses were performed using the SPSS version 25.0 statistical program.

**Results:**

The caries prevalence rates based on ICDAS II codes 1–6, 1–2, and 3–6 were 98.01%, 96.9%, and 85%, respectively. A statistically significant difference was observed among the different islands regarding the cutoff point for ICDAS II codes 3–6 (*p* ≤ 0.001). Participants aged 6–11 years had the highest caries prevalence.

**Conclusions:**

The results show a high prevalence of dental caries among children in the Galapagos Islands, which increases with age. Contrary to expectations, the study did not find a significant correlation between the severity of dental caries and the presence of dental biofilm.

## 1. Introduction

Dental caries is a significant global health issue, affecting a large portion of the population [1–4]. The World Health Organization (WHO) reports that 3.5 billion people have caries in their permanent teeth, while 530 million people have caries in their primary teeth [5–7]. In Latin America and the Caribbean, caries affects 90% of pediatric patients [8].

Traditionally, dental caries prevalence studies have used a classic evaluation system of the WHO [9–11], which determines whether dental surfaces are carious or not [9]. However, this system does not consider the different stages of the lesion and has limited sensitivity [11, 12]. The introduction of the International Caries Detection and Assessment System (ICDAS) has provided a more precise and reliable diagnosis, allowing for the identification of early stages of caries lesions. This has improved epidemiological reporting and clinical practice [12, 13].

In Ecuador, like many other countries around the world, the healthcare system is divided between the public and private sectors [14, 15]. This is also the case in the Galapagos Islands. However, there have been only two national epidemiological studies conducted in Ecuador [16, 17]. The first conducted in 1996 showed that 88.2% of the population under 15 years of age had carious lesions [16]. This percentage had a slight decrease in a second study carried out in 2009 where the prevalence reached 79.4% in 6-year-old children [17]. The Galapagos Islands are part of the Ecuadorian territory and have a significant number of inhabitants, despite this there is no report on the prevalence of caries in this area.

To address this knowledge gap, the Galapagos Oral Health Study (ESSO-Gal) was initiated on the islands of San Cristobal, Santa Cruz, and Isabela [18]. The objective of this article is to report the prevalence of dental caries in schoolchildren aged 2- to 11-year-old and evaluate the association between different carious lesions, dental biofilm, age, and island of origin. The ICDAS II criteria [19–21] were used for caries detection, while the simplified Silness–Löe index was used to indicate the presence of dental biofilm.

## 2. Methods

This study's data were sourced from the ESSO-Gal cohort, conducted between 2018 and 2020. The methodology and design of the ESSO-Gal cohort have been documented in a previously published protocol [18].

This research is a cross-sectional study that aimed to determine the prevalence of dental caries among children in the Galapagos Islands. The study also aimed to assess the relationship between dental caries and a range of factors, including the type of carious lesions, the presence of dental biofilm, the age of the participants, and their specific island of residency.

To calculate the sample size, the total school population of the Islands, 2,243 children, was taken into account. Using the Grandaria Mostral statistical program (GRANMO), a sample size of 837 individuals was estimated, with a 95% confidence level and a 0.03 margin of error. The assumed population percentage was set at ∼50%, with an additional 20% accounted for to accommodate nonresponses or attrition among participants. The sample was distributed among educational units on the islands of Santa Cruz, Isabela, and San Cristobal.

Boys and girls between the ages of 2 and 11 years who attended the selected educational units on the islands of San Cristobal, Santa Cruz, and Isabela in the Galapagos Archipelago were invited to participate in the study. The children's participation was subject to the signature of an informed consent form by their parents or legal representatives, as well as the informed consent of the children themselves. Only children who were legally enrolled in the educational units and who showed acceptable behavior according to the Frankl scale (scale for assessing patient cooperation), such as scale 3 and 4, were included. Patients with chronic, degenerative diseases or metabolic syndromes were excluded from the study [18].

To ensure participant anonymity, each child was assigned an identification code before undergoing a visual clinical examination of the oral cavity. The examiners had received prior training and were standardized in the use of these scales, as outlined in the protocol [18].

The presence of dental biofilm was assessed using the simplified Silness–Löe index, [19] which involved the examination of buccal or palatal/lingual surfaces. This examination covered either permanent central incisors (teeth 1.1 or 2.1 and 3.1 or 4.1) or primary teeth (teeth 5.1 or 6.1 and 7.1 or 8.1), as well as permanent first molars (teeth 1.6, 2.6, 3.6, and 4.6). In cases where the latter were not present, second primary molars (teeth 5.5, 6.5, 7.5, or 8.5) were examined. The assessment was conducted using a WHO probe on the tooth surfaces [22].

Following the examination, oral hygiene procedures, including dental brushing, were performed, and the removal of dental biofilm was verified. The diagnosis of carious lesions was made based on the ICDAS II criteria [20–23]. At the end of the diagnostic process, each participant received motivational activities, instructions for brushing using the modified Bass” technique, and an oral hygiene kit. Data were recorded in individual files and subsequently compiled in Excel spreadsheets, with the participants' numerical codes used to maintain anonymity.

### 2.1. Statistical Analysis

The statistical analysis was conducted using SPSS version 25.0. Descriptive statistics, including frequencies, percentages, measures of central tendency, and measures of dispersion, were used to describe the categorical and quantitative variables. Parametric and nonparametric measures were used depending on the distribution of the data.

The main study variable was caries according to the ICDAS II criteria. Three cutoff points were established on the scale: 1–6 (caries), 1–2 (noncavitated caries), and 3–6 (cavitated caries) [24–26]. Caries prevalence was determined using the Clopper–Pearson test for all cutoff points. Caries was analyzed both as a dichotomous variable (caries experience) and as a count variable (amount of caries per surface) using various statistical tests, including chi-square tests, Fisher's exact test, Mann–Whitney *U* test, and univariate negative binomial regression to establish initial associations.

Multivariate analyses were performed using robust Poisson regression models for caries experience and negative binomial regression models for amount of caries. Models were adjusted for age group, sex, island, and dental biofilm. Effect modifications and modifications in *P* values were also considered among all variables. By employing these statistical analyses, the study aimed to identify associations between dental caries and the studied factors while accounting for potential confounders and effect modifiers.

## 3. Results

### 3.1. Demographic Characteristics and Distribution of Subjects

In total, 804 participants were included in the study. Of these, 201 were residents of San Cristobal, 478 were from Santa Cruz, and 126 were from Isabela Islands. The overall prevalence of dental caries was 98.01%, with only 1.9% of children having healthy dentition. For the cavitated lesion category, the prevalence was reduced to 85%, while the remaining 15% of subjects were either caries free or were affected with noncavitated lesions. On the other hand, 96.9% of individuals presented at least one noncavitated lesion and 3.1% were either caries-free or presented only cavitated lesions.

When examining prevalence on the islands, San Cristobal had rates of 98.5% in dental caries, 89.6% in cavitated lesions, and 98.0% in noncavitated lesions. Similarly, individuals from Santa Cruz had prevalence rates of 97.7%, 88.5%, and 95.9% in the same categories. Isabela exhibited rates of 99.2%, 64.3%, and 99.2%, respectively.  Table 1 shows all the demographic components of the population.

Regarding the amount of caries per subject, measures of central tendency and dispersion, such as the mean and standard deviation, were presented based on demographic variables. Additionally, regression estimates for the dental biofilm index were calculated (Table 2).

### 3.2. Association Analysis of the Study Variables and Caries Categories

Table 1 also shows the association between participant characteristics and the various caries categories in the study. A statistically significant association was observed between age and all caries categories (*p*=0.001), while no statistical association was found for the sex variable. Regarding the island of origin, a statistically significant difference was evident within the cavitated lesions group (*p* < 0.001). Similarly, the difference between the biofilm index and caries experience was statistically significant.

### 3.3. Multivariate Models Analysis

Regarding the association between caries experience and the study variables, age showed statistical significance in all caries categories. In Isabela Island, children decrease the risk of caries of cavitated lesion in 29%. When the model was adjusted, there were no great changes observed in comparison with the unadjusted model (Table 3).

With respect to the association between the amount of caries per subject and the different demographic variables, it could be observed that the 6–11-year-old group presented higher risk of number of caries (in the categories of caries and noncavitated lesions) when compared with 2–5-year-old group. However, there was no statistical significance regarding the age group and the cavitated lesions category. Additionally, females had a lower risk of caries than males in all caries categories.

When analyzing the island of origin among inhabitants of Isabela Island, it was observed that the risk of developing cavitated lesions was 57% lower compared to San Cristobal inhabitants (Table 3).

The dental biofilm index demonstrated statistical significance in the caries group (ICDAS 1–6) and the noncavitated lesions group (ICDAS 1–2). However, no association was found between the biofilm index and the cavitated caries category (ICDAS 3–6). In the adjusted model, noncavitated surfaces showed no statistical differences based on sex and dental biofilm. This, however, changed for dental biofilm and the number of cavitated surfaces (*p*=0.02).  Table 4 shows all the values in detail.

Multivariate models of stratified analysis by age group are shown in Table 5. In the adjusted model, the biofilm variable demonstrated an association with dental caries and cavitated lesions categories. Within the 6- to 11-year-old group, only age did not exhibit statistical differences in the noncavitated lesion category. Additionally, females showed a lower prevalence of both cavitated and noncavitated lesions compared to males.

Decreased risk for caries was seen in Isabela individuals than in San Cristobal individuals. In the same way, for every unit of increase on the dental biofilm index, the risk of developing dental caries lesions increases by 13% and 23% for cavitated lesions category (Table 5).

## 4. Discussion

The findings of this study indicate a high prevalence of caries in the Galapagos Islands, with a significant number of both cavitated and noncavitated lesions. In fact, 98.01% of the participants presented some form of caries lesion. Similar high prevalence rates ranging from 81% to 93% have been reported in other rural areas of Ecuador [27–29], which aligns with the results of this study.

However, the prevalence results obtained in this study differ from other studies conducted in Ecuador and Latin America, where the figures decrease to around 60% [30]. Furthermore, when compared to developed countries, where caries prevalence ranges from 39.9% to 50%, the difference becomes even more pronounced [31, 32]. This variation in prevalence rates could potentially be explained by factors such as limited access to water sources with adequate fluoride levels [33], restrictions on the consumption of healthy foods [34], and a lack of healthcare personnel in the Galapagos Islands.

Regarding age, the study found a significant association between caries experience and the number of carious lesions. The risk of developing caries increased with age, although variations were also observed at different age groups [35]. While it is reasonable to assume that longer exposure time of the dental structure to an acid environment increases the risk of caries [2, 36], it has been noted that recently erupted teeth are more susceptible to caries than those with longer eruption time [37]. This may be attributed to enamel maturation [38], which stabilizes the risk of caries to some extent with increasing age. However, in the multivariate model of this study, no association was found between age groups and the number of cavitated caries. It suggests that behavioral factors beyond age may have a more significant influence on the development of carious lesions [39].

In terms of geographic location, Santa Cruz Island had the highest prevalence of caries. This outcome may be associated to the geographic and administrative conditions of the archipelago. Santa Cruz Island lacks drinking water, which is attributed to its poor water quality, and this absence of potable water [40], which is a risk factor for dental caries, could encourage unhealthy dietary practices [41, 42]. On the other hand, Isabela Island showed a protective factor in the experience of cavitated caries. This phenomenon may be attributed to the distinctive dietary preferences of its inhabitants, which differ significantly from those on the other two islands. Being one of the least densely populated islands in the archipelago and the one farthest from continental Ecuador, Isabela Island fosters dietary habits that involve reduced consumption of highly processed foods, emphasizing a diet enriched with natural fluoride sources like fish and seafood [43]. Furthermore, Isabela Island's considerable distance from the Ecuadorian mainland, coupled with trade restrictions, contributes to this unique dietary pattern [41, 42].

Previous studies have established an association between caries and dental biofilm (dental plaque) [44–46]. The presence of cariogenic bacteria, such as *Streptococcus mutans*, in dental biofilm leads to acid production, which causes demineralization of the tooth structure [47]. However, the demineralization process can be stopped even when there is a cavitary lesion, as long as the biofilm is controlled [48]. The results of this study support these mechanisms.

In addition, the cavitated caries experience did not show an association with dental biofilm in this study, and it is possible that the lesions reported with ICDAS II codes 3–6 had been restored. Consequently, the accumulation of biofilm on restored surfaces might differ from that on cavitated surfaces [49–51]. This limitation should be considered when interpreting the study's findings. Additionally, the study found differences in the number of caries based on the participants' sex, which is consistent with findings from other studies [52, 53].

As the first investigation of its kind conducted in the Galapagos Islands, this study provides relevant data on oral health in the archipelago. The high prevalence of caries calls for concern among health authorities in the country. One proposed solution is to implement oral health campaigns with active involvement from parents, teachers, and health personnel such as pediatric dentists in the islands, focusing on monitoring the school population [54–58].

The study has certain limitations. The age groups included individuals with both primary and mixed dentition [59–61], although some studies have reported differences in the risk of caries between primary and permanent teeth, while others have found no significant differences [62, 63]. Furthermore, while the study primarily evaluated the prevalence of caries, future studies on the island should consider other oral diseases such as periodontitis and gingivitis. Variables such as different age groups, limited access to oral healthcare on the island, socioeconomic conditions, and individuals' diets should be considered in future research.

## 5. Conclusions

In conclusion, this study revealed a high prevalence of dental caries, which increases with age. Santa Cruz Island showed the highest prevalence among the geographic areas studied. The presence of dental biofilm was high; however, it was not found to be associated with the presence of different codes of carious lesions.

These findings highlight the urgent need for oral health interventions and preventive measures in the Galapagos Islands. It is essential to develop targeted strategies for different age groups, as the risk of caries increases with age. Additionally, it is necessary to involve different stakeholders, such as parents, teachers, and health professionals, to address the high prevalence of dental caries and promote oral health among the population.

## Figures and Tables

**Table 1:** Demographic characteristics of the population and their association with the caries experience (ICDAS values 1–6, 1–2, and 3–6).

	Caries experience (ICDAS 1–2) *n* (%)			Caries experience (ICDAS 3–6) *n* (%)			Caries experience (ICDAS 1–6) *n* (%)		
	No	Yes	*p*	No	Yes	*p*	No	Yes	*p*
General	25 (3.1)	779 (96.9)		121 (15)	683 (85)		15 (1.9)	789 (98.1)	
Age (years)									
2–5	1.5 (1.9)	206 (25.6)	**0.001** ^**a**^	49 (6.1)	172 (21.4)	**0.001** ^**a**^	10 (1.2)	211 (26.2)	**0.001** ^**a**^
6–11	10 (1.2)	573 (71.3)		72 (9)	511 (63.6)		5 (0.6)	578 (71.9)	
Sex									
Male	11 (1.4)	382 (47.5)	0.68^a^	59 (7.3)	334 (41.5)	0.98^a^	9 (1.1)	384 (47.7)	0.38^a^
Female	14 (1.7)	398 (49.4)		62 (7.7)	350 (43.5)		6 (0.7)	406 (50.4)	
Island									
San Cristobal	4 (0.5)	197 (24.5)	0.09	21 (2.6)	180 (22.4)	**<0.001** ^**a**^	3 (0.4)	198 (24.6)	0.48^b^
Santa Cruz	20 (2.5)	458 (56.9)		55 (6.8)	423 (52.5)		11 (1.4)	467 (58.09)	
Isabela	1 (0.1)	125 (15.5)		45 (5.6)	81 (10.1)		1 (0.1)	125 (15.5)	

	Median (SD)	Median (SD)		Median (SD)	Median (SD)		Median (SD)	Median (SD)	

Dental biofilm index	1.05 (0.67)	1.55 (0.57)	**<0.001** ^**c**^	1.52 (0.58)	1.54 (0.58)^c^	0.72^c^	0.8 (0.48)	1.55 (0.57)	**<0.001** ^**c**^

^a^Chi-square test. ^b^Fisher's exact test ^c^Mann–Whitney *U* test. Bold values signify *p*-value < 0.05.

**Table 2:** Mean and standard deviation of the number of caries and their association with study variables.

Variable subgroup	All sample			Age group 2–5 years			Age group 6–11 years		
	ICDAS 1–2	ICDAS 3–6	ICDAS 1–6	ICDAS 1–2	ICDAS 3–6	ICDAS 1–6	ICDAS 1–2	ICDAS 3–6	ICDAS 1–6
	Mean (SD)	Mean (SD)	Mean (SD)	Mean (SD)	Mean (SD)	Mean (SD)	Mean (SD)	Mean (SD)	Mean (SD)
Age (years)									
2–5	9.7 (7.3)	7.1 (10.0)	16.8 (13.5)	9.7 (7.3)	7.1 (10.0)	16.8 (13.5)	13.7 (9.6)	7.3 (7.2)	21.1 (13.0)
6–11	13.7 (9.6)	7.3 (7.2)	21.1 (13.02)						
Sex									
Male	13.4 (9.9)	7.9 (8.6)	21.3 (14.2)	9.6 (7.6)	7.4 (10.8)	17.0 (14.4)	14.6 (10.3)	8.1 (7.7)	22.8 (13.8)
Female	11.9 (8.4)	6.7 (7.5)	18.6 (12.2)	9.7 (7.1)	6.8 (9.4)	16.5 (12.8)	12.8 (8.7)	6.6 (6.6)	19.5 (11.9)
Island									
San Cristobal	11.7 (8.0)	8.2 (6.9)	19.9 (12.03)	10.0 (7.2)	7.6 (8.6)	17.7 (13.2)	12.4 (8.2)	8.5 (5.9)	21.0 (11.3)
Santa Cruz	12.4 (9.5)	7.8 (8.8)	20.3 (13.96)	8.0 (6.9)	7.8 (11.5)	15.8 (14.4)	13.9 (9.8)	7.9 (7.7)	21.8 (13.5)
Isabela	14.8 (9.6)	3.5 (5.8)	18.4 (12.69)	13.9 (7.2)	4.1 (6.7)	18.1 (11.4)	15.3 (10.6)	3.2 (5.3)	18.5 (13.2)

	Estimate	Estimate	Estimate	Estimate	Estimate	Estimate	Estimate	Estimate	Estimate

Dental biofilm index^a^	0.10 ^*∗*^	0.12	0.10 ^*∗*^	0.12	0.25	0.18	0.05	0.06	0.05

^a^Bivariate negative binomial regression; ∗*p* < 0.05.

**Table 3:** Poisson regression model of caries experience based on ICDAS 1–6,1–2, and 3–6 cutoff points.

Variable subgroup	Caries experience (ICDAS 1–6)				Caries experience (ICDAS 3–6)				Caries experience (ICDAS 1–2)			
	Unadjusted model		Adjusted model^a^		Unadjusted model		Adjusted model^a^		Unadjusted model		Adjusted model^a^	
	RR (CI)	*p*	RR (CI)	*p*	RR (CI)	*p*	RR (CI)	*p*	RR (CI)	*p*	RR (CI)	*p*
Age (years)												
2–5	1	**0.02**	1	**0.04**	1	**0.003**	1	**0.02**	1	**0.006**	1	**0.01**
6–11	1.34 (1.00–1.06)		1.02 (1.00–1.05)		1.12 (1.00–1.21)		1.09 (1.01–1.18)		1.05 (1.01–1.09)		1.04 (1.01–1.08)	
Sex												
Male	1	0.53	1	0.37	1	0.89	1	0.99	1	0.59	1	0.78
Female	1.00 (0.98–1.02)		1.00 (0.99–1.02)		0.99 (0.94–1.05)		1.00 (0.94–1.06)		0.99 (0.97–1.01)		0.99 (0.97–1.02)	
Island												
San Cristobal	1		1		1		1		1		1	
Santa Cruz	0.99 (0.98–1.03)	0.57	1.00 (0.97–1.02)	0.8	0.99 (0.93–1.04)	0.72	0.77	0.77	0.98 (0.95–1.00)	0.17	0.99 (0.96–1.02)	0.58
Isabela	1.00 (0.97–1.01)	0.54	1.00 (0.98–1.03)	0.55	0.71 (0.62–0.84)	**<0.001**	0.71 (0.61–0.81)	**<0.001**	1.01 (0.98–1.03)	0.34	1.00 (0.98–1.03)	0.62
Dental biofilm index	1.03 (1.01–1.06)	**0.002**	1.03 (1.01–1.05)	**0.002**	1.01 (0.96–1.06)	0.65	1.06 (1.00–1.12)	**0.02**	1.04 (1.01–1.07)	**0.003**	1.03 (1.00–1.06)	**0.02**

RR, relative risk; CI, confidence interval at 95%; *p*, *p*-value. ^a^Model adjusted for age group, sex, island, and biofilm. Bold values signify *p*-value < 0.05.

**Table 4:** Negative binomial regression model and the number of cavities per surface, taking into account the ICDAS 1–2, 1–6, and 3–6 cutoff points.

Variable subgroup	Number of caries (ICDAS 1–6)				Number of caries (ICDAS 3–6)				Number of caries (ICDAS 1–2)			
	Unadjusted model		Adjusted model^a^		Unadjusted model		Adjusted model^a^		Unadjusted model		Adjusted model^a^	
	RR (CI)	*p*	RR (CI)	*p*	RR (CI)	*p*	RR (CI)	*p*	RR (CI)	*p*	RR (CI)	*p*
Age (years)												
2–5	1	**<0.001**	1	**0.002**	1	0.71	1	0.55	1	**<0.001**	1	**<0.001**
6–11	1.25 (1.11–1.41)		1.20 (1.07–1.35)		1.03 (0.84–1.27)		0.94 (0.77–1.14)		1.41 (1.26–1.58)		1.41 (1.26–1.58)	
Sex												
Male	1	**0.003**	1	**0.009**	1	**0.03**	1	**0.008**	1	**0.02**	1	0.07
Female	0.87 (0.79–0.95)		0.88 (0.80–0.97)		0.84 (0.72–0.98)		0.82 (0.7–0.95)		0.89 (0.80–0.98)		0.91 (0.83–1.01)	
Island												
San Cristobal	1		1		1		1		1		1	
Santa Cruz	1.02 (0.92–1.13)	0.64	1.04 (0.93–1.18)	0.43	0.96 (0.86–1.12)	0.61	1.06 (0.88–1.28)	0.49	1.07 (0.95–1.20)	0.25	1.03 (0.92–1.17)	0.60
Isabela	0.92 (0.79–1.03)	0.28	0.89 (0.77–1.03)	0.11	0.43 (0.31–0.58)	**<0.001**	0.38 (0.29–0.51)	**<0.001**	1.27 (0.09–1.47)	**0.001**	1.26 (1.09–1.46)	**0.002**
Dental biofilm index	1.11 (1.02–1.21)	**0.008**	1.13 (1.03–1.25)	**0.007**	1.13 (0.98–1.29)	0.07	1.06 (1.00–1.12)	**0.02**	1.10 (1.01–1.20)	**0.02**	1.04 (0.95–1.14)	0.39

RR, relative risk; CI, confidence interval at 95%; *p*, *p*-value. ^a^Model adjusted for group age, sex, island, and biofilm. Bold values signify *p*-value < 0.05.

**Table 5:** Negative binomial regression model and the number of cavities per surface based on the caries experience.

Variable subgroup	Number of caries (ICDAS 1–6)				Number of caries (ICDAS 3–6)				Number of caries (ICDAS 1–2)			
	Unadjusted model		Adjusted model^a^		Unadjusted model		Adjusted model^a^		Unadjusted model		Adjusted model^a^	
	RR (CI)	*p*	RR (CI)	*p*	RR (CI)	*p*	RR (CI)	*p*	RR (CI)	*p*	RR (CI)	*p*
Group age 2–5 years												
Age	1.22 (1.06−1.41)	**0.005**	1.29 (1.12−1.50)	**0.001**	1.26 (0.94−1.69)	0.11	1.32 (1.03−1.70)	**0.03**	1.19 (1.04−1.37)	**0.01**	1.32 (1.14−1.53)	**<0.001**
Sex												
Male	1	0.86	1	0.86	1	0.64	1	0.98	1	0.96	1	0.81
Female	0.98 (0.79−1.21)		0.98 (0.79−1.21)		0.91 (0.62−1.33)		0.99 (0.70−1.41)		0.99 (0.81−1.21)		0.97 (0.79−1.19)	
Island												
San Cristobal	1		1		1		1		1		1	
Santa Cruz	0.90 (0.70−1.15)	0.42	1.08 (0.82−1.44)	0.92	1.03 (0.70−1.51)	0.86	1.18 (0.78−1.79)	0.43	1.38 (1.09−1.75)	**0.01**	1.61 (1.23−2.10)	**<0.001**
Isabela	1.02 (0.78−1.33)	0.87	0.98 (0.76−1.28)	0.58	0.54 (0.30−0.96)	**0.04**	0.47 (0.27−0.81)	**0.007**	0.80 (0.63−1.01)	0.07	0.85 (0.67−1.08)	0.19
Dental biofilm index	1.20 (0.98−1.46)	0.06	1.26 (1.02−1.57)	**0.03**	1.29 (0.93−1.79)	0.12	1.62 (1.16−2.27)	**0.005**	1.13 (0.95−1.34)	0.15	1.07 (0.89−1.28)	0.46
Group age 6–11 years												
Age	0.90 (0.85−0.96)	**0.002**	0.99 (0.96−1.03)	0.63	0.90 (0.85−0.96)	**0.002**	0.93 (0.87−0.98)	**0.01**	1.03 (0.99−1.08)	0.06	1.03 (0.99−1.07)	0.16
Sex												
Male	1	**0.002**	1	**0.002**	1	**0.01**	1	**<0.001**	1	**0.02**	1	**0.04**
Female	0.85 (0.77−0.94)		0.85 (0.77−0.94)		0.82 (0.70−0.96)		0.76 (0.65−0.89)		0.87 (0.78−0.98)		0.89 (0.80−1.00)	
Island												
San Cristobal	1		1		1		1		1		1	
Santa Cruz	1.04 (0.93−1.16)	0.47	1.10 (0.98−1.24)	0.77	0.93 (0.79−1.08)	0.36	1.04 (0.87−1.24)	0.67	1.11 (0.98−1.27)	0.10	1.14 (0.99−1.31)	0.06
Isabela	0.88 (0.74−1.05)	0.17	0.86 (0.72−1.02)	0.11	0.38 (0.26−0.55)	**<0.001**	0.37 (0.26−0.52)	**<0.001**	1.22 (1.02−1.47)	**0.03**	1.18 (0.98−1.42)	0.08
Dental biofilm index	1.05 (0.97−1.151)	0.18	1.13 (1.03−1.24)	**0.01**	1.06 (0.93−1.20)	0.36	1.23 (1.05−1.43)	**0.008**	1.05 (0.95−1.16)	0.26	1.08 (0.97−1.20)	0.15

RR, relative risk; CI, confidence interval at 95%; *p*, *p*-value. ^a^Model adjusted for age, sex, island, and biofilm. Bold values signify *p*-value < 0.05.

## Data Availability

The data that support the findings of this study are available from the corresponding author upon reasonable request.
